# The Human Skin-Derived Precursors for Regenerative Medicine: Current State, Challenges, and Perspectives

**DOI:** 10.1155/2018/8637812

**Published:** 2018-07-11

**Authors:** Ru Dai, Wei Hua, Heng Xie, Wei Chen, Lidan Xiong, Li Li

**Affiliations:** ^1^Department of Dermatology, West China Hospital, Sichuan University, No. 37, Guo Xue Xiang, Chengdu, Sichuan 610041, China; ^2^Department of Dermatology, Ningbo First Hospital, Zhejiang University, No. 59, Liuting Street, Ningbo, Zhejiang 315010, China

## Abstract

Skin-derived precursors (SKPs) are an adult stem cell source with self-renewal and multipotent differentiation. Although rodent SKPs have been discussed in detail in substantial studies, human SKPs (hSKPs) are rarely reported. Understanding the biological properties and possible mechanisms underlying hSKPs has important implications for regenerative medicine particularly clinical applications, as human-derived sources are more suitable for clinical transplantation. The finding that hSKPs derivatives, such as neural and mesodermal progeny, have both *in vitro* and *in vivo* function without any genetical modification makes hSKPs a trustable, secure, and accessible resource for cell-based therapy. Here, we provide an overview of hSKPs, describing their characteristics, originations and niches, and potential applications. A comparison between traditional and innovative culture methods used for hSKPs is also introduced. Furthermore, we discuss the challenges and the future perspectives towards the field of hSKPs. With this review, we hope to point out the current stage of hSKPs and highlight the problems that remain in this field.

## 1. Introduction

Stem cells (SCs) are a type of cells with self-renewal and multilineage differentiation capacities. Mounting studies have suggested the existence of SCs in somatic tissues, including the bone marrow, adipose tissue, and skin [1, 2]. The skin is of particular appeal to researchers because of its diverse advantages. The average area of an adult skin is 1.75 m^2^ [3], providing adequate sources for research and transplantation. In addition, the skin is a complex tissue which contains various SCs [4], like epidermal SCs, dermal SCs, and hair follicle-derived SCs, making it a good candidate for regenerative medicine. Moreover, skin-derived SCs are easily accessible with minimal invasion, less pain, and quick recovery.

Skin-derived precursors (SKPs), firstly described by Toma et al. in 2001 [5], are a novel kind of SCs from the dermis. SKPs grow as floating spheres, in the suspension serum-free medium supplemented with basic fibroblast growth factor (bFGF) and epidermal growth factor (EGF), similar to neurosphere, and they can self-renew and have multilineage differentiation capacities. Excitingly, SKPs display the characteristics of neural SCs for expressing Nestin and generating neural progenies, consequently becoming competitive SCs for regenerative medicine, especially in a neurological field. Rodent SKPs have been extensively discussed in substantial literature. Despite sharing some properties with human SKPs (hSKPs) [5, 6], the rodent ones are inappropriate to mimic hSKPs *in vivo* and *in vitro* due to species differences. Moving toward clinical applications, it is crucial to characterize the biological properties and the *in vivo* features of hSKPs. In this review, we aim to provide representative information about hSKPs regarding their biology, potential applications, challenges, and perspectives based on reported works.

## 2. What Are hSKPs?

### 2.1. The Notion of hSKPs

hSKPs are a kind of self-renewal and multipotential precursor population from a human dermis [6]. In a similar manner to neural SCs, hSKPs express neural-related proteins and generate neural progenies. Once being exposed to certain environments, hSKPs can contribute to all three primary germ layers. Although hSKPs are known for neural SCs-like properties, they have many advantages over neural SCs. On one hand, isolation of neural SCs requires a high invasive procedure, while hSKPs were obtained with a minimal invasion, as they are located superficially in the skin. On the other hand, the yield of neural SCs is limited to the tissue supply, whereas the skin is a large organ covering the whole body, providing abundant sources for hSKPs. Both hSKPs and dermal mesenchymal SCs are obtained from the skin. However, they are different in characteristics. Firstly, as distinct from dermal mesenchymal SCs, hSKPs grow and expand as spheres in medium containing bFGF, EGF, and B27, conditions optimized for neural SCs, while dermal mesenchymal SCs are cultured in an adherent condition with serum. Secondly, dermal mesenchymal SCs are mostly used in skin disorders like skin atrophy or skin repair [7, 8]. Although previous studies have indicated that SKPs are capable of antiaging [9, 10], the most promising aspects of hSKPs are the possibility that they can provide an accessible, abundant, multipotent source for nervous system disorders such as spinal cord injury.

Here, we provided a comparison of current stem cells with potential application in the field of nervous system disorders (Table 1).

### 2.2. The Profiles of hSKPs

hSKPs were first reported to express Nestin, fibronectin, and vimentin [5, 6]. Similar studies were repeated in other laboratories later, and markers expressed in hSKPs were reported to be different in different groups. At present, there are no specific markers identified in hSKPs, which greatly prevent hSKPs from purification and further characterization. We summarized the reported expressive markers for hSKPs in Table 2. hSKPs were phenotypically distinct from melanocytes and haematopoietic SCs, as they were negative for tyrosinase [6], c-kit [18], and CD34 [19]. However, it was noteworthy that several recent studies indicated that hSKPs from different anatomical regions showed varied expression profiles and levels [20, 21]. It is important to realize that hSKPs cultured as floating spheres were heterogeneous, composed of a diversity of cells. As reported by Etxaniz et al., a subpopulation of p75 neurotrophin receptor (NTR)^+^CD56^+^ dermospheres from hSKPs that retained neural competence belonged to the Schwann cell lineage in an adult human dermis [22]. These neurogenic cells are derived from Schwann cell dedifferentiation of specialized Schwann cells at the peripheral nerve ending in the skin rather than resident precursor cells. Terminal Schwann cells could also behave as bona fide dermal stem cells. Gresset et al. [23] and Iribar et al. [24] also revealed the existence of the stem cell-like populations within adult neural crest cells in the skin, which could also be cultured as floating spheres. Generally speaking, these results suggest that spheres obtained from floating culture are actually a complex mixture of diverse cells, including stem, precursor, and terminally differentiated cells, while it is still unclear to what extent to which the diversity affected the biotechnological applications of hSKPs. There are also no markers available to distinguish purified hSKPs from other cells currently.

### 2.3. Differentiation of hSKPs

#### 2.3.1. Neurogenesis

The neurogenesis of hSKPs is particularly attractive. When cultivated in neurobasal medium supplemented with neurotrophic factors such as nerve growth factor, brain-derived neurotrophic factor, and NT-3, hSKPs underwent a sequential change and exhibited neuron-like morphology and expressed neural marker *β*III-tubulin [6]. These neurons were mostly peripheral in nature as they expressed p75NTR, a marker for peripheral neurons. It was now reported that some hSKPs-derived neurons even expressed a mature central neuronal marker of the K^+^-Cl^+^ cotransporter KCC2 [30]. Cocultured with primary murine glia, hSKPs-derived neurons could generate action potentials. Some neurons even presented the synaptic marker of synapsin and generated synaptic activities [30]. When exposed to hippocampal astrocyte-derived signals, these neurons could also generate voltage-dependent calcium transients [31]. In addition to neurons, hSKPs could differentiate into glial cells of Schwann cells [6] and astrocytes [32]. As for glial cell differentiation, under the condition of neurobasal medium containing N2 supplement, forskolin, and heregulin *β*, we could observe a bipolar morphological population expressing S100*β* and/or p75NTR and/or CNPase and/or glial fibrillary acidic protein (GFAP) [6]. These hSKPs-derived Schwann cells were capable of inducing myelination and expressing myelin-related protein when coculturing with embryonic rat dorsal root ganglion neurons *in vitro* [33].  Table 3 shows the detailed protocols for neurogenesis.

#### 2.3.2. Other Lineages

Other ectodermal differentiations of hSKPs have also been reported. hSKPs were capable of generating pigmented melanocytes in the presence of SCs factor and endothelin-3. Such kind of melanocytes could migrate from the dermis to the epidermis and be located individually in the basal layer of the epidermis [35]. The differentiation of hSKPs into corneal endothelial cells (CECs) was achieved through coculturing with B4G12 cells. These hSKPs-derived CECs presented polygonal morphology and expressed a corneal endothelial major marker of Na^+^/K^+^ ATPase [36].

What about mesodermal cell types? hSKPs under multi-inducting conditions have resulted in adipocytes [6], osteoblasts [37], chondrocytes [38], and other cell types [25]. Adipogenesis is ascertained through lipid accumulation in the cytoplasm and expression of adipogenic markers. Osteogenesis is evaluated by calcium deposit, the higher alkaline phosphatase (ALP) activity level, and the elevated expression of osteogenic proteins or genes. Chondrogenesis is measured by forming cartilage-like micromasses, emergence of sulfated glycosaminoglycan content, and detection of cartilage-related proteins or genes. As for smooth muscle cell formation, it is also confirmed by expression of specific markers. We summarized the representative methods used for mesodermal differentiation of hSKPs in Table 4.

The differentiation of hSKPs has extended to endodermal cells. Mehrabi and his colleagues successfully differentiated hSKPs into glucose-responsive, insulin-producing clusters [39]. When exposed to hepatogenic factors and cytokines, hSKPs can cross the lineage border and generate hepatocyte-like cells [40]. The differentiation capacity of hSKPs is more plastic than expected, and the comprehensive function of hSKPs should be explored in future experiments.

## 3. Where Do hSKPs Originate and Locate?

### 3.1. The Origination of hSKPs

The initial isolation of hSKPs was based on the hypothesis that the skin contained cells with neurogenic properties. The capacity of hSKPs to generate both neuronal and glial cells confirmed this hypothesis. However, while hSKPs appeared to be similar to neural SCs in their culture behavior and neural phenotypes, they also expressed mesodermal markers like fibronectin, which raised the question about their developmental origins.

Neural crest contributes a wide variety of tissues, including diverse cells in the skin [41]; the mainstream concept considered hSKPs to be endogenous neural crest precursors that arose in the skin during embryogenesis and persisted into adulthood [16, 18]. The hypothesis was supported by the following evidences: firstly, when plating single cells dissociated from foreskin samples directly and adherently, these dermal cells were able to generate neurons, glial cells, and smooth muscle cells without any passaging and expansion [6]. Secondly, an analysis of hSKPs gene expression also revealed that they expressed a variety of neural crest-associated transcription factors including Pax3, Snail, and Slug [6]. Thirdly, studies on the rodents provided more detailed evidence. As described by Fernandes et al., the transplanted SKPs were able to follow the host neural crest migratory pathways. Moreover, SKPs from Wnt1-Cre/R26R transgenic mice, which selectively expressed reporter transgenes in cells of crest origin, expressed reported transgenes [18].

Another alternative possibility was that hSKPs represented a heterogeneous sphere containing terminally differentiated neural crest cells. Under certain condition and exposure to growth factors, hSKPs experienced a dermal disaggregation procedure that allowed them to cellularly dedifferentiate and acquire multipotency. For example, it has recently been reported that the neurogenic property of hSKPs was retained exclusively by a subgroup of p75NTR^+^CD56^+^ spheres that, presenting characteristics of the Schwann cell lineage, resulted from Schwann cell dedifferentiation. Such *in vitro* reprogramming procedures involved the regulation of the expression level of Sox2 [22]. An early study by Wong et al. demonstrated that multipotent neural crest-derived terminal cells from the skin also displayed self-renewal capacity and could be grown and expanded in a floating sphere culture. Moreover, in the trunk skin, spheres presenting neural crest stem cell-like properties were restricted to the glial and melanocyte lineages rather than mesenchymal origination [42]. Another two recent studies provided further evidence to support that neurogenic potential detected in the sphere culture was ascribed to the neural crest-derived population of the Schwann cell lineage instead of dermal multipotent precursors [23, 24]. Together, these data challenged the hypothesis whether hSKPs reflected an endogenous precursor with multipotent potential, as neural lineages were suggested to arise from neural-derived Schwann cells. Therefore, due to the conflicting evidence, it was currently unclear if hSKPs may give rise to neural derivatives [33, 43]. Obviously, the precise originations of hSKPs remains to be seen.

### 3.2. The Niches of hSKPs

Historically, hSKPs were initially described to be from adult human scalp, suggesting that human scalp contains SKPs, but their precise niche had not been determined. To address this question, Fernandes and his colleagues performed a further study in 2004, indicating that dermal papillae of hair and whisker follicles were niches for rodent SKPs [18]. hSKPs might share the same region as rodent ones. Based on this hypothesis, Hunt et al. microdissected the dermal papilla of adult human skin specifically and obtained sphere-forming cells [26]. Although the initial cell seeding number was lower, there was no surprise that more spheres and better expansion were observed in microdissected dermal papilla samples from dermal papillae in contrast to the whole skin. The fate-mapping approach combined with enriched papillasphere generation demonstrated that dermal papillae of hair follicles constituted a niche for hSKPs.

Toma et al. challenged their previous concept that dermal hair follicles were probably not the only niches for hSKPs by harvesting spheres from neonatal foreskin, a tissue that does not contain hair follicles. The foreskin-derived spheres expressed similar markers to hSKPs from the scalp and generated three germ layer progenies [6], raising the question: where do the glabrous skin-derived hSKPs come from? German scholars utilized the flow cytometry technique to acquire a pure population of nonfollicular hSKPs. And they found hSKPs resided in the papillary layer of the dermis and the dermal perivascular regions represented a niche for these cells. These cells were CD146-positive and showed no morphological or functional differences (adipogenic and osteogenic differentiation) with their follicular counterparts [20]. However, Etxaniz et al. demonstrated that these CD146^+^ perivascular cells failed to yield neural progeny in the presence of neurogenic condition [22].

Recently, hSKPs are successfully isolated from dermal fibroblast culture, indicating that dermal matrix may be another niche for hSKPs. A study conducted by Wenzle et al. developed the dermal fibroblast to spherical cells cultured from patients with Hutchinson-Gilford progeria syndrome [25]. Particularly, in this study, researchers adopted low temperature as a stress to screen hSKPs residing in dermal fibroblasts. These temperature-resistant cells showed similar features as primary hSKPs. Moreover, in their recent study, they performed acidic stress on dermal fibroblasts prior to hSKPs isolation and got a similar finding [44]. However, in another group, they directly isolated hSKPs from precultured monolayer fibroblasts of both normal and diseased adult dermises without any stressors [34]. Such fibroblast-derived hSKPs under differential conditions also led to lipid accumulation, calcification, and S100*β* or *β*III-tubulin expression.

**Figure 1:**
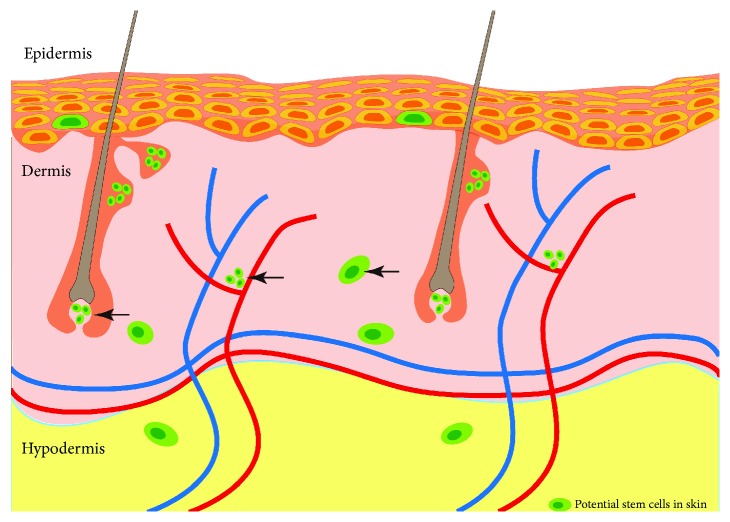
A cross section of human skin and the potential stem cells residing in them. Arrows denote the niches for hSKPs: dermal papillae, perivascular regions, and dermal matrix.

At present, the precise localization responsible for hSKPs remains unidentified. It seems that multiple niches contribute to hSKPs generation (Figure 1). However, it should be acknowledged that the majority of what qualifies themselves as “hSKPs” are currently considered the dedifferentiation of “terminally differentiated” cell. For example, mature Schwann cells derived from the skin could undergo dedifferentiation and displayed SKP-like characteristics in response to isolation and culture conditions [45, 46]. Therefore, the novel definition of niches for hSKPs probably represents the location of these terminally differentiated cells rather than skin-derived multipotent precursors.

## 4. How to Get hSKPs?

### 4.1. Isolation and Expansion of hSKPs

**Figure 2:**
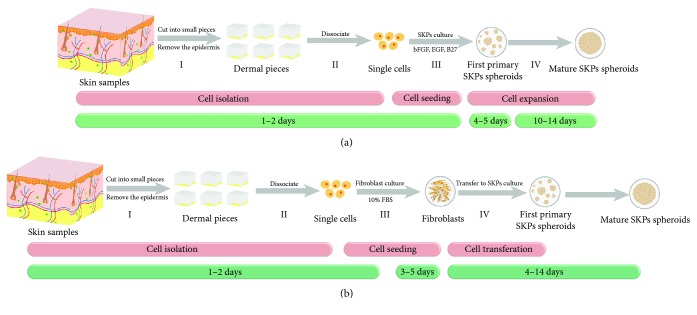
The protocols for hSKPs culture. (a) Traditional method to culture hSKPs: (I) skin samples are cut into pieces, and the epidermis is removed after enzymatic digestion. (II) The pieces are mechanically dissociated into single cells. These two steps are known as cell isolation, and they usually take 1-2 days for isolation procedure. (III) Single cells are seeded into SKPs medium containing bFGF, EGF, and B27, and primary spheroids with small size will be observed 4-5 days after initial seeding. (IV) The spheres can be expanded for 10–14 days with mature colonies of large, phase bright, and spherical clusters. (b) Transferred method to culture hSKPs: (I) skin samples are cut into pieces, and the epidermis is removed after enzymatic digestion. (II) The pieces are mechanically dissociated into single cells. (III) Single cells are seeded into fibroblast medium with 10% FBS, and mature fibroblast colonies with spindle shape will be observed after 3–5 days. (IV) Cells from fibroblast culture are transferred into SKPs culture as the traditional procedure. After 4–14 days, mature spheres will be observed. SKPs: skin-derived precursors; hSKPs: human skin-derived precursors; bFGF: basic fibroblast growth factor; EGF: epidermal growth factor; FBS: fetal bovine serum.

hSKPs can be harvested from both hairy and glabrous human skins. The current protocol to isolate a single cell requires enzymatic digestion and mechanical trituration (Figure 2(a)) [47]. Typically, one human neonatal foreskin sample will yield approximately 10^7^ cells while only 0.01% to 0.001% of the total cells are hSKPs [27]. The growth of hSKPs is highly related to cell density and culture conditions [2, 28]. Generally, the lower the cell density, the slower their growth. However, a density higher than 25,000 cells/ml may result in cell aggregation instead of proliferating clusters. As for culture conditions, it is essential to grow hSKPs in the medium with the conditioned medium supernatant from the last passage. The recommended medium for prompting hSKPs expansion and passaging is composed of 50% fresh medium and 50% conditioned medium, while the saved conditioned medium should be filtered through a sterile filer before using [4].

Age may be an important factor for hSKPs yield. A study collected foreskin-derived hSKPs from 102 healthy subjects aged from 8 months to 85 years and found a negative correlation of sphere-forming potential with age. The samples from subjects over 50 years old lost sphere-forming ability. hSKPs from younger subjects have a larger size of dermospheres and better differentiation potential than those from elderly ones [48]. However, it is clearly known that the aged dermis becomes atrophic and it is relatively acellular, avascular, and less nervous. The density of hair follicle also goes through a progressive reduction with age [49]. Therefore, the depletion might be attributed to a reduction of hSKPs niches in the aged dermis rather than spherogenic difference in samples from a different age. While another study by Yoshikawa et al. successfully isolated hSKPs from all ages, even in individuals from 70 to 78 years old [21], together these data conflict the effect of age on hSKPs and whether the donor age is associated with cellular yield will be on considerable interest. Anatomical location is another factor. Gago et al. analyzed the derivation and phenotype of hSKPs from the foreskin, scalp, breast, and abdomen. The results proved that all area could produce hSKPs in spite of a higher yield from the foreskin. However, the scalp-derived hSKPs were positive for p75NTR and foreskin-derived hSKPs were negative for versican [48]. Yoshikawa et al. supported this result, but they argued that facial skin generated more hSKPs and exhibited a high differentiation capacity into neural and mesodermal cells than those of the trunk or extremity skin [21]. Interestingly, a recent study by Kwon et al. compared the variability of tissue-engineered constructs with different anatomical locations and observed distinctive characteristics in construct formation. When using an engineered cartilage as a model, constructs formed from foreskin and abdominal skin-derived hSKPs displayed similar mechanical properties at implantation and remained comparable after *in vivo* culture, while breast skin-derived constructs were out of the mechanical test, though all groups were proved to be biocompatible [50]. Together, these data suggest that a further application of hSKPs should take the anatomical location into consideration.

### 4.2. New Methods for Establishing hSKPs

The present method to establish hSKPs has many drawbacks. Firstly, only a few thousands of hSKPs can be acquired from traditional protocols, significantly limiting their further clinical applications. Secondly, the entire procedure to generate hSKPs is complicated and time-consuming. Generally, the isolation procedure takes at least one day and the whole expansion requires one to three weeks. Thirdly, hSKPs adhere to the plastic easily during incubation while premature adherence will greatly reduce the yield of hSKPs.

Traditionally, hSKPs are cultured in a conventional two-dimensional (2D) environment, which is inappropriate to mimic an *in vivo* cellular environment. As a result, three-dimensional (3D) scaffolds mimicking the nature niche in the body are utilized to grow hSKPs. Wang et al. compared the morphology, growth property, standard gene expression, and differentiation potential of hSKPs grown in routine suspension condition and different 3D hydrogels. All 3D hydrogels could support hSKPs growth, while the hyaluronic acid-based scaffold facilitated hSKPs proliferation highly and retained their characteristics better [51]. 3D condition-derived hSKPs showed delaying senescence in long-term culture *in vitro*, which is a common drawback for most somatic SCs. Another study investigated the biological property of hSKPs along with different nanofibrous scaffolds. After being seeded into the scaffolds, all hSKPs extended the process and covered the surface of nanofibers gradually. These hSKPs remained vigorous and proliferative, while enhancing production of extracellular matrix and sulphated glycosaminoglycans in nanofibrous scaffolds [52]. Stirred suspension bioreactors have also been reported to facilitate hSKPs yield. hSKPs expanded higher in agitation condition than in static cultures, especially at the rate of 60 rpm [28].

Since hSKPs are known for similar to neural SCs, which can be expanded as neurospheres or as adherent monolayers, several studies tried to grow hSKPs in monolayers. Earlier in 2004, Joannides et al. investigated to grow hSKPs in mitogens followed by attachment conditions in serum. The sequential altering culture could increase cell expansion, while retaining inducible neural potential [31]. Later, two independent groups tried to isolate multipotent hSKPs from primary monolayer fibroblast culture [34, 37, 44], as illustrated in Figure 2(b). Spheres from this transferred method also displayed SKP property. Currently, a study seeded dissociated dermal cells into a serum-supplemented culture first and then transferred into a serum-free adherent condition reinforced by growth factors. Growing hSKPs by the permanent adherent culture would not change their protein expression and differential potentials [53]. However, the identity of monolayer-derived hSKPs remained ambiguous. Scholars proposed those cells as dermal mesenchymal SCs rather than traditional hSKPs [54], since dermal mesenchymal SCs were also known for self-renewal ability and neurogenic potential [55, 56]. Induction of hSKPs from induced pluripotent SCs (iPSCs) is another hotspot. Sugiyama-Nakagiri et al. firstly cultured human iPSCs with recombinant noggin and SB431542, an inhibitor of activin/nodal and transforming growth factor- (TGF-) *β* signaling, and then transferred to SKP medium containing CHIR99021, a WNT signal activator. The authors efficiently induced hSKPs from iPSCs with an induction rate over 97%, and iPSC-derived hSKPs expressed characteristic genes and proteins of hSKPs as well as differentiated into adipocytes, osteoblasts, and Schwann cells [55]. Nevertheless, optimization of protocols for hSKPs isolation and differentiation into potential clinical application remains a challenging prerequisite to reach clinical translation.

## 5. Possible Mechanism to Regulate hSKPs' Stemness

A number of articles noticed that hSKPs lost their SCs properties gradually in the long-term expansion. Liu et al. found a dramatic decline of Akt activity of hSKPs during isolation and passage process [56]. Akt, also known as protein kinase B, is the downstream signal of the phosphatidylinositol 3-kinase- (PI3K-) Akt (PI3K-Akt) pathway. The PI3K-Akt pathway plays a key role in controlling cell proliferation, migration, survival, and quiescence, especially in neural SCs [57]. Since hSKPs were established for exhibiting similar properties as neural SCs, Liu et al. focused on the PI3K-Akt pathway as a candidate for maintaining hSKPs function. To further confirm the hypothesis, authors blocked this pathway with several inhibitors to find that hSKPs senescence increased and self-renewal decreased consequently. On the contrary, enhancing the PI3K-Akt pathway improved self-renewal of hSKPs and alleviated their senescence [56]. To understand the possible mechanism in detail, Liu et al. continued the research. They performed a time-dependent microarray analysis of hSKPs cultured *in vitro* and found that the genetic pattern of senescent hSKPs was different from typical senescence. A subsequent investigation into cyclin-dependent kinase inhibitor genes pointed out p15^INK4b^ and p27^KIP1^ might be involved in hSKPs senescence. The next analysis of upstream signals suggested Akt and Forkhead box O3 (FOXO3) hypoactivity contributed to hSKPs senescence. Activation of Akt and blockage of FOXO3, p15^INK4b^, and p27^KIP1^ reversed the senescent condition of hSKPs and facilitated hSKPs proliferation. Therefore, this study initiatively proposed the role of Akt-FOXO3- p15^INK4b^/p27^KIP1^ in regulating hSKPs' behavior [58].

## 6. Therapeutic Potential of hSKPs

The great plasticity held by hSKPs indicates their potential application in cell-based treatment for several diseases, although conflictive evidence suggests that some of the proposed potentials may be attributed to in vitro expansion conditions.

As a requisite to translational applications, however, it is essential to examine the biological safety and immunocompatibility of hSKPs and to demonstrate that hSKPs represent a secure source. Using a G-banding set-up, the karyotype of hSKPs was shown to maintain a normal state even after passaging for 15 months [6]. As for immunocompatibility, Kock et al. illustrated that hSKPs expressed HLA-ABC molecule rather than HLA-DR, suggesting that they were poorly immunogenic. When exposed to proinflammatory stimulation, the immunophenotype of hSKPs was not changed as well, indicating that even in the inflammatory condition, hSKPs could be considered a source with poor immunogenicity as well. In addition, to be poorly immunogenic, hSKPs possessed immunosuppressive features. In fact, hSKPs could downregulate the expression of the lymphocyte costimulatory molecules on stimulated T cells and inhibit the activation and proliferation of allogeneic activated peripheral blood mononuclear cell both *in vivo* and *in vitro*. Being transplanted into severe combined immunodeficiency disease (SCID) mice, hSKPs significantly impaired the graft-versus-host response and allowed a longer survival time [19]. Therefore, hSKPs were poorly immunogenic and could modulate the allogeneic immune response. Cellar contact and secretion of soluble inhibitory factors involved the immune modulation procedure. However, Le et al. proposed the rodent SKPs, through the loss of Nf1, to be the cellular origination of NF1-associated dermal neurofibroma [59]. In support of this hypothesis, another human study by Iribar et al. found Schwann cell precursor marker CDH19 and Schwann cell dedifferentiation marker Sox2 were significantly upregulated in neurofibromas from diagnostic patients. They posited that the dedifferentiation of Schwann cells played a role in human dermal neurofibroma development, while dedifferentiated Schwann cells were suggested to form hSKPs [60]. Taken as a whole, further work is needed in order to illuminate clear understanding of hSKPs biological safety, although most studies concluded hSKPs as “a safe grafting source.”

The neural potentials held by hSKPs *in vivo* suggest they might be a cell-based treatment for neurological diseases. At two months after engrafting hSKPs into lateral right ventricles in SCID mice, these cells migrated to the frontal cortex, striatum, and hippocampus. At striatal levels and in host cortex, they generated cells with a neuronal phenotype. In the frontal cortex, the most accumulated was donor-derived astrocytes. These cells efficiently incorporated into white matter regions of hosts and extended processes to adjacent blood vessels in the frontal cortex. No donor-derived oligodendrocytes were found in host brains [32]. It was firstly reported that hSKPs could not only survive but also hold their differentiation after transplantation, which provided a solid base for their further application. In 2014, Krause et al. directly transplanted hSKPs-induced Schwann cells into sciatic nerves of SCID mice with local demyelination. Four days later, many human cells could be detected in the transplant region with some aligning along axons. At 30 days after transplant, some human cells were showed to express S100*β* and aligned along myelin sheaths, consistent with a myelinating Schwann cell phenotype [33]. Another study supported that transplantation of hSKPs into shiverer mice brain could result in compact myelin, a substance considered to be absent in the central neural system of shiverer mice, which suggested the generation of functional Schwann cell after grafting [61]. Thus, hSKPs are a promising treatment for the spinal cord injury (SCI). The pathophysiology of SCI is caused by gradual loss of neurons, axons, and glial cells after primary or secondary injury process. Another potential application is for Hirschsprung disease, a common neurocristopathy disease in human neonates due to incomplete colonization of enteric ganglion cells. hSKPs were able to differentiate into enteric neurons and glia. An ex vivo gut explant assay showed mouse SKPs colonized and differentiated into Ret^k−/k−^ aganglionic hindgut [62]. Although the study did not investigate hSKPs function in ex vivo explants, it is more likely that a similar phenomenon can be expected in human counterparts.

Shen et al. found hSKPs could generate functional CECs *in vivo* and treat corneal endothelial dysfunction. By injecting these hSKPs-derived CECs into the rabbit and monkey corneal endothelial dysfunction models, the thickness of the cornea decreased significantly and the clarity of the cornea improved greatly in the treatment group. At the 7th day after injection, the corneas became clearly transparent. Confocal microscope images also showed that Descemet's membrane was covered by hSKPs-derived CECs with a polygonal shape [36]. Besides ectodermal cell types, hSKPs could also generate skeletogenic cell types, contribute to bone repair [38], and differentiate into adipocytes, chondrocytes, hepatocytes, pancreatic cells, and other cells.

Although mounting evidence has illustrated hSKPs properties in the laboratory, no clinical trial of hSKPs has been reported so far according to the official clinical trial website (https://clinicaltrials.gov/) and no studies have been reported on humans. Therefore, more work is needed to be done about hSKPs before being translated into clinical practice. One of the most interests of further work is the possibility of using functional derivatives such as Schwann cells and neuronal cells in therapeutic and reparative strategies for clinical disorders in human.

## 7. Challenges and Future Perspectives

Although hSKPs are good candidates for regenerative medicine because of their rich source, easy acquisition, and multipotency, they are still far from an “off the shelf” product since a number of problems remain unsolved. Firstly, the optimal method to culture hSKPs with a large quantity and simple procedure has not been well established. Although some innovative protocols have been brought forward to enhance hSKPs, these protocols are far from widespread application. Secondly, as little progress has been made on investigating the properties of hSKPs in the body *in vivo*, their long-term behavior in the body remains unknown. To fully recognize their biosafety and efficiency, more studies should be performed on that part. Thirdly, the fact that hSKPs are heterogeneous spheres composing diverse cells makes them of great need for purification, as it is still unknown whether the heterogeneity will affect hSKPs function in clinical applications or not. Finally, the possible mechanisms involved in regulating hSKPs function are not demonstrated thoroughly. However, understanding the underlying mechanisms is more essential for further clinical application. In the near future, hSKPs may become the mainstay of cell-based treatment for various diseases.

## Figures and Tables

**Table 1:** Comparison of stem cells for nervous system disorders.

Category	Source	Advantages	Disadvantages	Ref.
Embryonic stem cells	Embryonic tissues or developing nervous system	Pluripotency, nonimmunogenic	Insufficient sources, ethical problems, possible tumorigenesis	[11, 12]
Induced pluripotent stem cells	Somatic cells	Pluripotency, no ethical problems, sufficient sources	Unstable induction, possible infection	[11, 13]
Adult neural stem cells	Adult neural tissue	Multipotency, no ethical problems	Insufficient sources, hard acquisition, purified problems	[11, 14]
SKPs	Dermis	Multipotency, no ethical problems, sufficient sources, easy acquisition	Relatively low neurogenic cells, purified problems	[15, 16]
Mesenchymal stem cells	Including bone marrow, adipose tissue	Multipotency, no ethical problems, sufficient sources	Relatively low neurogenic cells, purified problems, relatively hard acquisition	[11, 17]

**Table 2:** Summary of the relevant markers expressed by hSKPs.

Category	Markers	Ref.
Neural crest stem cells or neural system-related markers	Nestin, p75NTR, Pax3, Snail, Slug, Msx1, Twist, Lamin A/C, Dermo-1, Musashi, Sca-1, Sox9, Sox10, S100*β*	[5, 6, 19, 21, 25–27]
Mesenchymal or dermal cell-related markers	Fibronectin, vimentin, versican, Collagen III, *α*SMA	[4–6, 27, 28]
Embryonic stem cell-related markers	Sox2, Oct4, TG30, Nanog	[4, 25]
Endothelial, perivascular, or stromal cell-related markers	CD90, CD95, CD105, CD146	[19, 20]
Costimulatory molecule-related markers	CD40	[19]
Cell adhesion molecule-related markers	CD29, CD 44, CD49e, CD 54, CD166	[19]
Immunoregulatory molecule-related markers	CD73, HO-1	[19]
Human leukocyte antigen-related markers	HLA-ABC, intracellular HLA-G	[19]
Oncogenesis and development-related markers	Wnt-5a	[21]
Cellular proliferation-related markers	Ki67	[29]

*α*SMA: α smooth muscle actin; HLA: human leukocyte antigen; NTR: neurotrophin receptor.

**Table 3:** The protocols for neurogenesis of hSKPs.

Differentiation	Basic medium	Reagent (concentration)	Indication	Ref.
Neuron differentiation	Neurobasal or DMEM/F12 (3 : 1) medium	Brain-derived neutrophic factor (50 ng/ml), nerve growth factor (50 ng/ml), neurotrophiin-3 (10 ng/ml), FBS (1%–5%)	Differentiation for 2–4 weeks with 50% of the medium changed every 3-4 days	[6]
Schwann cell differentiation	Neurobasal or DMEM/F12 (3 : 1) medium	N_2_ supplement (1% or 2%), forskolin (4 *μ*m or 5 *μ*m), heregulin*β* (10 ng/ml or 50 ng/ml), FBS (1%)	Differentiation for 2–4 weeks with 50% of the medium changed every 3-4 days	[6, 34]
Astrocyte differentiation	B27 neurobasal medium	FBS (3%–10%)	Differentiation for 3 weeks with 50% of the medium changed every 3-4 days	[32]

DMEM: Dulbecco's modified Eagle medium; FBS: fetal bovine serum.

**Table 4:** The protocols for mesodermal and endodermal differentiation of hSKPs.

Differentiation	Basic medium		Reagent (concentration)	Indication	Ref.
Adipocyte differentiation	DMEM medium		IBMX (0.45 nM), insulin (2.07 *μ*M), dexamethasone (100 nM), amphotecticin B (0.5 *μ*g/ml), Pen/Step (100 units/100 *μ*g/ml), rabbit serum (15%)	Differentiation for 2-3 weeks with medium changed every 3-4 days	[34]
Osteocyte differentiation	DMEM medium		Dexamethasone (100 nM), *β*-glycerophosphate (10 mM), L-ascorbic acid 2-phosphate (50 *μ*M), Pen/Step (1%), FBS (10%)	Differentiation for 2-3 weeks with medium changed every 3-4 days	[34]
Chondrocyte differentiation	DMEM/F12 (3 : 1) medium		Dexamethasone (100 nM), ascorbic acid (250 mM), bone morphogenetic protein-2 (50 ng/ml), Pen/Step (1%), FBS (10%)	Differentiation for 2-3 weeks with medium changed every 2-3 days	[38]
SMC differentiation	DMEM medium		PDGF-BB (5 ng/ml), TGF-*β*1 (2.5 ng/ml), FBS (5%)	Differentiation for 3-4 weeks with medium changed every 3 days	[25]
Insulin-producing cell differentiation	Stage 1	High-glucose DMEM/F12 (1 : 1) medium	EGF (20 ng/ml), bFGF (40 ng/ml), B27 (1%), final glucose (17.5 mM)	Culture until forming spheres	[39]
	Stage 2	Low-glucose DMEM medium	db-cAMP (1 mM), RA (1 *μ*M), B27 (1%), 2% FBS, final glucose (5 mM)	Culture for 2 days	
	Stage 3	High-glucose DMEM/F12 (1 : 1) medium	Nicotinamide (10 mM), insulin-like growth factor 1 (10 mM), activin-A (2 nM), B27 (1%), 2% FBS, final glucose (17.5 mM)	Culture for up to 1 week	
	Stage 4	As stage 3	As stage 3 (except without insulin-like growth factor 1)	Before transferring, cells are trypsinized and resuspended	
Hepatocyte differentiation	DMEM/F12 (3 : 1) medium		Day 0: FGF-4 (10 ng/ml) Days 1-2: FGF-4 (10 ng/ml), HGF (30 ng/ml) Days 3–5: FGF-4 (5 ng/ml), HGF (30 ng/ml), 0.5 × ITS Days 6–8: HGF (30 ng/ml), 0.25 × ITS, dexamethasone (20 *μ*g/l) Days 9–11: HGF (20 ng/ml), dexamethasone (20 *μ*g/l) From day 12 onwards: HGF (20 ng/ml), dexamethasone (20 *μ*g/l), OSM (10 ng/ml)	Differentiation for 3–6 weeks with medium changed every 3 days	[40]

DMEM: Dulbecco's modified Eagle medium; IBMX: 3-isobutyl-1-methylxanthine; FBS: fetal bovine serum; SMCs: smooth muscle cells; PDGF: platelet-derived growth factor; TGF: transforming growth factor; EGF: epidermal growth factor; bFGF: basic fibroblast growth factor; db-cAMP: dibutyryl cyclic adenosine monophosphate; RA: all-trans retinoic acid; FGF-4: fibroblast growth factor-4; HGF: hepatocyte growth factor; ITS: insulin-transferin-selenite; OSM: oncostatin M.

## Abbreviations

SCs: Stem cells
SKPs: Skin-derived precursors
bFGF: Basic fibroblast growth factor
EGF: Epidermal growth factor
hSKPs: Human skin-derived precursors
NTR: Neurotrophin receptor
HLA: Human leukocyte antigen
GFAP: Glial fibrillary acidic protein
CECs: Corneal endothelial cells
ALP: Alkaline phosphatase
2D: Two-dimensional
3D: Three-dimensional
iPSCs: Induced pluripotent stem cells
TGF: Transforming growth factor
FOXO3: Forkhead box O3
SCID: Severe combined immunodeficiency disease
SCI: Spinal cord injury
*α*SMA: *α* smooth muscle actin
DMEM: Dulbecco's modified eagle medium
IBMX: 3-Isobutyl-1-methylxanthine
FBS: Fetal bovine serum
SMC: Smooth muscle cells
PDGF: Platelet-derived growth factor.
